# Supplementary material for: Effects of weight-bearing dance aerobics on lower limb muscle morphology, strength and functional fitness in older women
Source: PeerJ. 2024 Jun 28;12:e17606. doi: 10.7717/peerj.17606 (PMC11216199; doi:10.7717/peerj.17606)
Supplement: Supplemental Information 2 [file peerj-12-17606-s002.docx]

The result of physical activity and nutrition intake

(mean±SD)

|  | intervention(n=18) | | control(n=15) | | Between-group | |
| --- | --- | --- | --- | --- | --- | --- |
|  | pre | post | pre | post | *F/χ²* | *P* |
| ST(min/d) | 424.68±204.82 | 420.90±131.49 | 391.52±180.15 | 406.00±146.74 | 0.04 | 0.84 |
| TPA(min/d ) | 328.32±113.98 | 368.27±113.62 | 332.71±120.63 | 336.05±87.01 | 1.30 | 0.26 |
| LPA(min/d ) | 287.23±100.67 | 277.63±108.79 | 278.52±123.32 | 309.00±105.34 | 0.72 | 0.40 |
| MVPA(min/d )  M(P_25_,P_75_) | 27.86  (0,65) | 38.57  (38.57,145.71) | 46.43  (0,105) | 0  (0,51.43) | -2.77 | **<0.01** |
| Calcium Supplements |  |  |  |  |  |  |
| Yes N(%) | 13(72.22%) | 13(72.22%) | 8(53.33%) | 11(73.33%) | 0.28 | 0.41 |
| Vitamin D |  |  |  |  |  |  |
| Yes N(%) | 3(16.67%) | 3(16.67%) | 5(33.33%) | 6(40%) | 0.03 | 0.63 |

Note：min/d: min/day；SB: sedentary behavior; TPA: total physical activity; LPA: light physical activity; MPA: moderate physical activity; VPA: vigorous physical activity

a: post vs. pre，*P*<0.05
