# Supplementary material for: Effects of weight-bearing dance aerobics on lower limb muscle morphology, strength and functional fitness in older women
Source: PeerJ. 2024 Jun 28;12:e17606. doi: 10.7717/peerj.17606 (PMC11216199; doi:10.7717/peerj.17606)
Supplement: Supplemental Information 4 [file peerj-12-17606-s004.docx]

# 自我报告的体力活动问题

**自我报告的体力活动问题**

一、个人基本信息

1. 姓 名：

2. 性 别：

3. 出生年月：

二、问卷调查

01. 通常情况下，您采用的交通方式及其时间

| 交通工具 | 每周平均几天 | 平均每天累计多少分钟 |
| --- | --- | --- |
| 乘车（船） |  |  |
| 自驾车 |  |  |
| 骑摩托车、电动车、助动车 |  |  |
| 骑自行车/共享单车 |  |  |
| 老年代步车 |  |  |
| 步行 |  |  |
| 其他，请注明____________ |  |  |

02.通常情况下，您的家务劳动情况

| 活动内容 | 每周平均几天 | 平均每天累计多少分钟 |
| --- | --- | --- |
| 小强度家务劳动（劳动时，与平时相比，吃力或疲惫感觉不明显，如擦桌、做饭等） |  |  |
| 中等强度家务劳动（劳动时，与平时相比，能感觉到吃力或出汗，如拖地、遛狗、园艺等） |  |  |
| 大强度家务劳动（劳动时，明显感觉到比平时吃力和疲惫，如搬（举）重物） |  |  |
| 其他家务工作，请注明____________ |  |  |

03. 您目前是否还工作（是指您的职业活动，包含农活或志愿者工作，但不包括平时的家务劳动）

①是 ②否**（请跳至第5 题）**

04. 通常情况下，您工作时的状态

| 活动内容 | 每周平均几天 | 平均每天累计多少分钟 |
| --- | --- | --- |
| 以静坐伏案为主（用电脑、书写等） |  |  |
| 工作中静坐并伴有上肢活动，或者以站为主（如司机、售票员、流水线组装工等） |  |  |
| 以走为主（如护士、卖场销售等） |  |  |
| 体力付出大（如人工搬运、举中午或挖掘、干农活等） |  |  |
| 其他活动，请注明____________ |  |  |

05. 通常情况下，您闲暇时间的静态活动情况

| 活动内容 | 每周平均几天 | 平均每天累计多少分钟 |
| --- | --- | --- |
| 看电视、电脑、手机、平板等活动 |  |  |
| 下棋、打牌、打麻将、练习书法、弹琴  等活动） |  |  |
| 读书、看报、听广播等活动 |  |  |
| 其他活动，请注明____________ |  |  |

06-1. 您是否进行体育锻炼？

①是 ②否**（请跳至第7 题）**

06-2. 通常情况下，您进行体育锻炼的情况（请先选择锻炼频次，再填写相应的锻炼时间）

| 活动内容 | 频次 | 平均**每次**累计多少分钟 |
| --- | --- | --- |
| 小强度体育锻炼（锻炼时呼吸和心跳平稳，和平常差不多，没有特别感觉，如散步等） | □每周不足1次  □每周1次及以上请注明_____次 |  |
| 中等强度体育锻炼（锻炼时呼吸与心跳加快但不急促，微微出汗，如快走、健步走、骑车等） | □每周不足1次  □每周1次及以上请注明_____次 |  |
| 大强度体育锻炼（锻炼时呼吸急促，心跳明显加快，出汗较多，如快跑、打球） | □每周不足1次  □每周1次及以上请注明_____次 |  |
| 其他锻炼，请注明____________ | □每周不足1次  □每周1次及以上请注明_____次 |  |

07. 您每周进行力量练习（包括俯卧撑、深蹲等徒手练习或使用哑铃、弹力带等健身器械的练习）的次数为：

① 0 次 ② 1 次 ③ 2-3次 ④ 4-5 次 ⑤ 6 次及以上

08. 您经常参加体育锻炼的项目（限选1-3 项，并按照重要程度排序）

经常： 次之： 再次：

① 走（散步、健步等各种走） ② 跑步

③ 游泳 ④ 骑车

⑤ 乒、羽、网球、柔力球等球类活动

⑥ 足球、篮球、排球等球类活动

⑦ 保龄球、地掷球、门球 ⑧ 健身路径

⑨ 体操（广播操、艺术体操、健美操等

⑩ 舞蹈（交际舞、体育舞蹈、民间舞蹈等）

⑪武术（武术套路、太极拳、太极剑、木兰扇等）

⑫ 格斗类（跆拳道、空手道、拳击、击剑等）

⑬ 气功（易筋经、八段锦等）、瑜伽

⑭ 力量练习（徒手、器械等） ⑮ 登山、攀岩等

⑯ 跳绳、踢毽 ⑰ 冰雪活动

⑱ 其他___________

09.您的钙摄入情况 ：

①摄入牛奶、钙片等

②不摄入

10.维生素补充情况：

①维生素D，如维生素D_2_、维生素D_3_

②不补充
