# Supplementary material for: Effects of weight-bearing dance aerobics on lower limb muscle morphology, strength and functional fitness in older women
Source: PeerJ. 2024 Jun 28;12:e17606. doi: 10.7717/peerj.17606 (PMC11216199; doi:10.7717/peerj.17606)
Supplement: Supplemental Information 5 [file peerj-12-17606-s005.docx]

Dear editor and reviewers,

I am writing to explain the addition of new authors to a submission.

After having a first decision, the addition of new authors to a submission is indeed rare event. Actually, when the first submission on November 11th, 2023, Peijie Chen was not in authors list. And on November 16th, 2023, we received a letter form *Peerj* about *changes required*, which mentioned “*Clinical Trial registration at https://www.chictr.org.cn/showprojEN.html?proj=127906 mentions Chen Peijie as study leader, but they are not an author. Please explain in a Note in the Confidential Information for PeerJ Staff.*” Considering the crucial role Peijie Chen played in trial registration, coordination, and communication with relevant institutions, we added Peijie Chen as author when we resubmitted on November 18th, 2023. This means that before the manuscript was assigned to an academic editor, and before the start of peer-review, Peijie Chen was already the author of this study. As for why Peijie Chen is still considered as a newly added author after this major revision, we are not quite sure as well.

We value academic norms and ethics highly and recognize the importance of accurately determining authorship for the fairness and credibility of academic outputs. The decision to include Peijie Chen as an author was based on a renewed recognition of his contributions and adheres to academic norms and ethics. And this changed been approved by all co-authors.

Thank you for your understanding and support. We look forward to continuing our collaboration with *Peerj* and jointly promoting academic research.

Sincerely,

Xiaoying Peng,

represented all co-author,

April 13, 2024.
